# Addressing transport safety and accessibility for people with a disability in developing countries: a formative evaluation of the Journey Access Tool in Cambodia

**DOI:** 10.1080/16549716.2018.1538658

**Published:** 2018-11-13

**Authors:** Julie A. King, Mark J. King, Niki Edwards, Sara A. Hair, Sarim Cheang, Anita Pearson, Sophie Coelho

**Affiliations:** a School of Public Health and Social Work (SPHSW), Institute for Health and Biomedical Innovation (IHBI), Queensland University of Technology (QUT), Kelvin Grove, Australia; b Centre for Accident Research and Road Safety - Queensland (CARRS-Q), Institute for Health and Biomedical Innovation (IHBI), Queensland University of Technology (QUT), Kelvin Grove, Australia; c Handicap International (now renamed Humanity and Inclusion), Phnom Penh, Cambodia

**Keywords:** audit, built environment, barriers, public transport

## Abstract

**Background**: The intersection between health, disability and transport has significant practical challenges for people with a disability living in low- and middle-income countries (LMICs), where road infrastructure is poor and travel unsafe. Lack of transport access to health, education, employment and other services impedes achievement of the Sustainable Development Goals and affects quality of life. The Journey Access Tool (JAT) combines access audit and road safety audit approaches to identify barriers to transport on journeys taken by people with a disability. To be useful and effective, it must fit the expectations of people with a disability (be acceptable) and be feasible for use in different settings (adoptable). Accordingly, a formative evaluation process was undertaken in Phnom Penh, Cambodia.

**Objectives**: To undertake a formative evaluation of the JAT using an iterative process to tailor the tool, pilot its use by people with a disability, and develop a template for its implementation in other LMICs.

**Methods**: An iterative process of consultation and three pilots was undertaken. Participants were people with a disability who undertook journeys with a public transport component accompanied by assistants. Focus groups were held after each pilot, and results were integrated into JAT revisions.

**Results**: Issues of terminology were resolved early, as were process issues related to the length of time taken to complete the JAT. Interpersonal issues were more difficult to address, with assistants tending to exceed their role and record their own comments. Use of the tool provided rich information on barriers.

**Conclusions**: The JAT was both acceptable and adoptable for people with a disability and other stakeholders, and the experience gained will facilitate adaptation of the tool to new settings. The tool has significant potential to shape and support advocacy for change and engagement with transport services and also health, education, employment and other services.

## Background

Disability is a significant public health issue internationally. It has been estimated that more than 15 per cent of the world’s population have a moderate or severe disability, with rates being higher in developing countries [1]. The Sustainable Development Goals (SDGs) highlight a number of development objectives for people with a disability [2]. Many, such as access to health care, employment and social interaction, rely on physical access and transportation. Research conducted by the first author in Thailand [3] found that transport for people with a disability living in a developing nation was considerably hampered by poor physical infrastructure unless they could be specially transported for the entire journey, which was prohibitively expensive for most people. In many low- and middle-income countries (LMICs) the road system often lacks footpaths, while those that are present are often in poor repair, blocked by vehicles, rubbish, vendors or roadside furniture, and lacking in ramps and other features that would assist people with a disability. In rapidly motorising countries it is often unsafe to cross the road because of poor compliance with traffic signals, crossing signs, lane/direction compliance and other rules, a high proportion of two- and three-wheeled vehicles and deteriorating road surfaces. These problems affect the safety and access of all people using the road, but present additional significant challenges to people with a disability [3], who may have a greater need for a smooth and continuous path, may have less speed when crossing roads, and may have sensory impairments that constrain their perception and prediction of emerging traffic situations. The achievement of the SDGs in relation to people with a disability therefore demands that these issues be addressed; however, the scale of the task appears overwhelming due to the many kinds of impairment people with a disability experience, and their intersection with the widespread physical infrastructure deficits outlined above.

**Figure 1.**
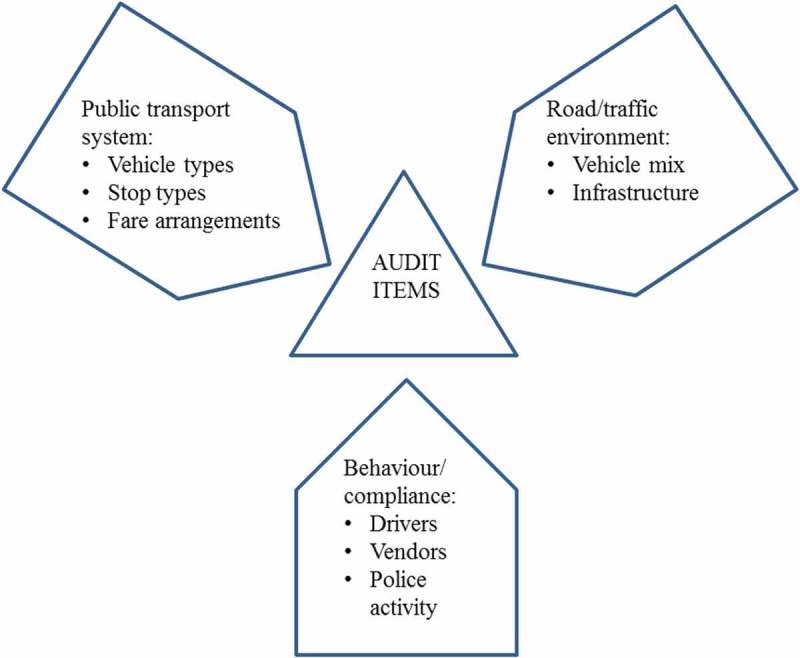
Conceptual basis of the JAT.

**Figure 2.**
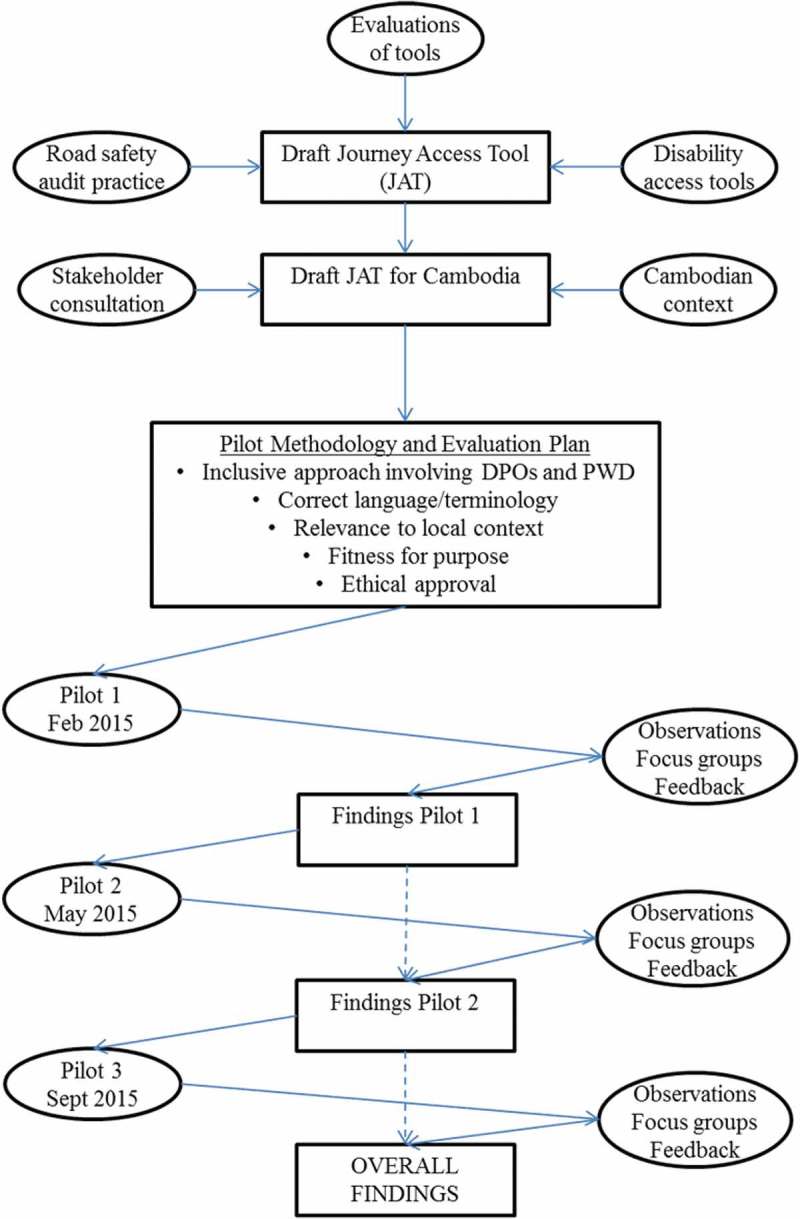
Overview of formative evaluation process.

**Figure 3.**
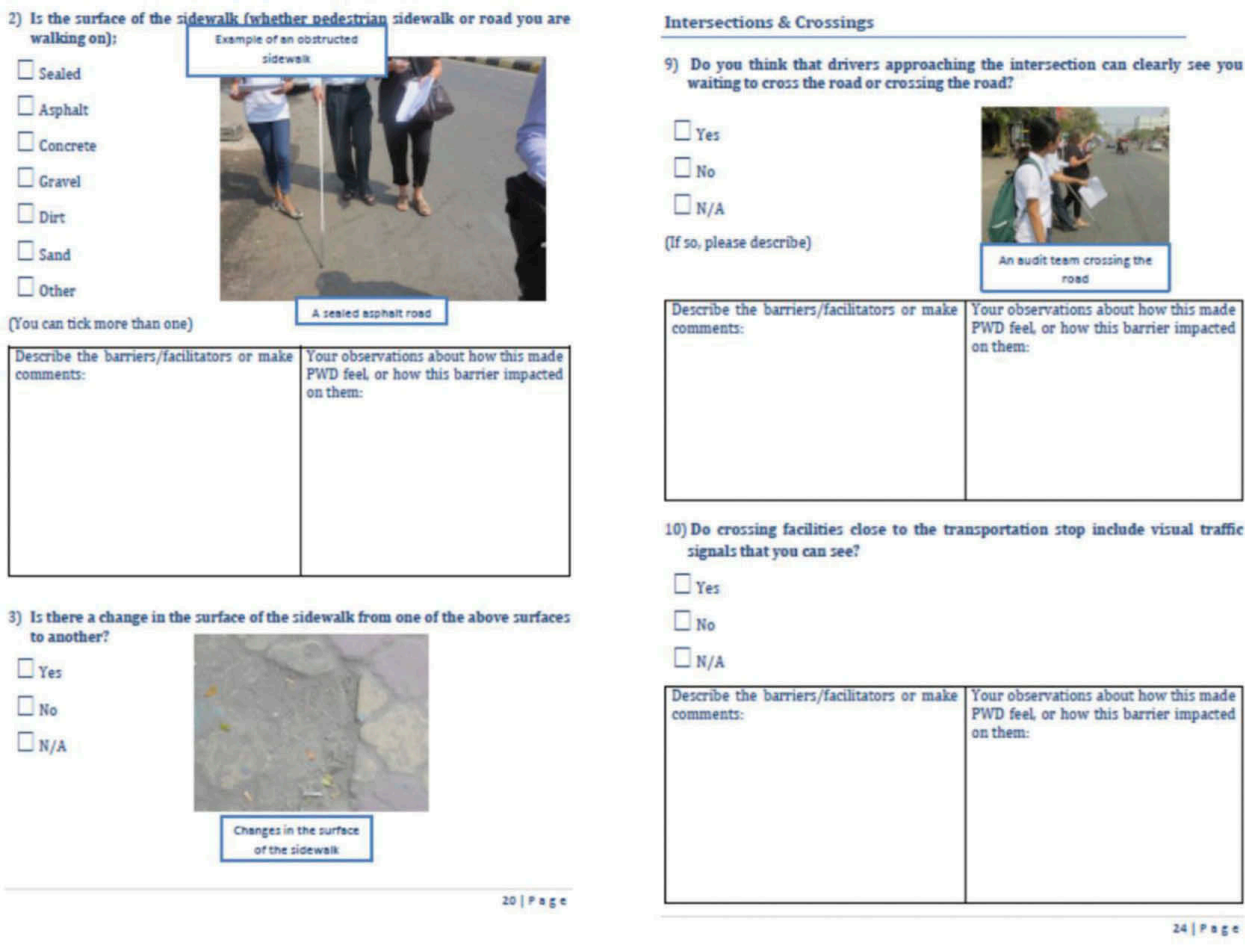
Sample pages from draft JAT.

Our team at Queensland University of Technology (QUT) developed a conceptual approach to addressing these issues in a manageable way that also incorporates direct engagement and feedback from people with a disability. The approach combines *access audits* and *road safety audits* into a *Journey Access Tool* (JAT), which is used for a personal ‘journey’ regularly taken by a person with a disability when they utilise services such as a hospital/clinic, employment or education, or seek general community access. *Access audits* are intended to provide governments, organisations and communities with a clear understanding of the accessibility of facilities, areas that require attention and recommendations for improvement [4]. Audit findings are incorporated into a structured plan of action, with highlighted priorities and achievable, realistic timeframes. *Road safety audits* of existing roads involve examination of a road section or intersection by a qualified and independent team, who compile a report on the road section’s safety, deficiencies, crash potential and potential resolutions [5]. A journey to access a service may involve using paths, crossing roads, taking public transport and accessing buildings; the factors that need to be taken into account in the JAT therefore fall into three categories: road and traffic environment; public transport system; and behaviour of other road users (Figure 1).

A search and examination of other audit tools and checklists for people with a disability was undertaken. Findings confirmed that the conceptual framework for the JAT was a unique approach (see Table 1).

**Table 1.** Other audit tools and checklists located and examined.

Source	Name
Apelt et al. [6]	Wayfinding system audit
Australian Federation of Disability Organisations [7]	Access to premises campaign (includes simple unnamed tool)
CAI Asia et al. [8]	Walkability audit reports
Canadian Transport Agency [9]	Reservation checklist for people with disabilities
CDC Healthy Aging Research Network and Easter Seals Project Action [10]	Neighborhood wayfinding assessment
Civil Rights Division of U.S. Dept of Justice [11]	Checklist for existing facilities
Civil Rights Division of U.S. Dept of Justice [12]	ADA checklist for polling places
Civil Rights Division of U.S. Dept of Justice [13]	ADA checklist for new lodging facilities
Civil Rights Division of U.S. Dept of Justice [14]	ADA checklist for emergency shelters
Corporation for National and Community Service [15]	Inclusion: Creating an inclusive environment: a handbook for the inclusion of people with disabilities in national and community service programs
Department of Transport: Western Australia [16]	Walkability audit tool
Disability Services Commissioner [17]	Good practice guide and self audit tool
Drum et al. [18]	Outpatient health care useability profile v4
Easter Seals and the CENTURY 21 System [19]	Easy access housing for easier living
Grant [20]	Access audit handbook
Government of Western Australia et al. [21]	Access resource kit: creating accessible communities with checklists to improve access for people with disabilities
Harkley et al. [22]	Accessible pedestrian signals: a guide to best practice
Health by Design [23]	How walkable is your neighbourhood?
Holdsworth-Wild et al. [24]	Disability access: A good practice guide for the arts
Martin [25]	Improving access to heritage buildings: a practical guide to meeting the needs of people with disabilities
National Health Service: Scotland [26]	Access audit survey toolkit: access for disabled people in healthcare premises
O’Fallon [27]	The public transport accessibility audit process
Pikora [28]	Survey of the physical environment in local neighbourhoods: spaces instrument observer’s manual
Roads and Traffic Authority NSW [29]	How to prepare a pedestrian access and mobility plan
Samarthyam National Centre for Accessible Environments [30]	Access audit report of Matri Mandir
Sandler [31]	Universal design and green home survey checklist
United Methodist Committee [32]	Accessibility mini-audit for churches
USA Access Board [33]	UFAS accessibility checklist
USA Department of Labor Employment and Training Administration [34]	Existing facilities checklist
USA Department of Transport: Federal Highway Administration [35]	Designing sidewalks and trails for access: Part II of II: Best practices design guide
Urban Management Department of the Lebanese Company for the Development and Reconstruction of Beirut Central District & UN ESC for Western Asia [36]	Access for the disabled: a design manual for a barrier free environment
Whittlesea Community Connections [37]	Whittlesea community engagement audit tool

The JAT has been specifically designed to be used by people with a disability, and potentially by disabled peoples’ organisations – i.e. the perspective of the user is prioritised. In this way, it differs from the road safety audit process, which is undertaken by independent professionals. However, it is similar to the road safety audit process in following the audit with a phase of discussion with responsible authorities about prioritisation of the issues identified.

## Objectives

To be useful in LMICs, the JAT needs to be adaptable to the local context and to be implemented using a process that accommodates the needs of people with a disability, who are the users. To this end, a formative evaluation was undertaken. The overarching objective was to undertake a formative evaluation of the JAT in Cambodia, where the relationship between the QUT team and Handicap International (HI, now Humanity and Inclusion) presented a collaborative opportunity that prioritised the views of people with a disability and other key stakeholders. More specific objectives were:

- To use an iterative formative evaluation process to tailor the tool to the Cambodian context;
- To pilot use of the tool by people with a disability, with assistance;
- To develop a template to inform implementation of the JAT in other contexts.

## Methods

Formative evaluation typically uses qualitative techniques to refine an intervention and improve its chances of success (e.g. Morrow [38]). A common objective in formative evaluation is to enhance the *acceptability* of a programme or intervention, where acceptability can be defined as ‘perceptions of (the program’s/intervention’s) design, strengths, weaknesses, cultural congruence, and value’ [38, p. 14]. It is a key component of the development and implementation of health interventions [39]. Among other criteria of acceptability is the degree to which it meets the needs of the target group within the particular setting [40]. This is closely related to a second objective of formative evaluation, which is to determine the *adoptability* of an intervention; this goes beyond acceptability to encompass feasibility of the intervention in the particular setting and in relation to the target group [41,42].

In terms of process, formative evaluation can be undertaken before or during implementation [43], and in this case was undertaken in both phases. Figure 2 presents an overview of the steps followed, in chronological order, including the initial process of concept development described in the Background. The COREQ guidelines for reporting qualitative research were followed.

A key part of the approach taken involved collaboration with HI, a large international non-governmental organisation that has undertaken programmes and advocacy strategies supporting empowerment and agency of people with a disability for several decades, in many parts of the world. In Cambodia, HI has also been involved in road safety programmes. HI Cambodia provided input into development of the JAT for the Cambodian context, recruited participants with a disability and their assistants using its network of volunteers and cooperating organisations such as schools and universities, and organised the three pilots of the JAT. QUT and HI team members together conducted the focus groups and observations, and provided reflective feedback on the processes. The senior members of the HI staff involved were mostly non-Cambodian (from France and the UK), while senior and mid-level Cambodian staff involved were fluent in English.

### Drafting of initial tool

The initial JAT items were developed to focus on: the quality of the built environment when used on a trip as a pedestrian or equivalent (e.g. a wheelchair user on a footpath) in terms of both access and safety; the ease and safety with which traffic on these trips can be negotiated; access to public transport stops and vehicles; and access to trip destinations. The specific items were based on examples of infrastructure and barriers that could be readily seen in and around Phnom Penh.

In the past it has been reported that most public transport systems in Cambodia are not accessible to people with a disability [44], and anecdotal evidence is that this remains a problem even though people with a disability are entitled to free public transport. The Law on the Protection and the Promotion of the Rights of Peoples with Disabilities 2009 was passed to increase the accessibility of transportation and meet international obligations under the United Nations Convention on the Rights of Persons with Disabilities [45]. However, there is little monitoring and enforcement of legislation, and as a result a limited amount of information is available about disability and access to transport within these contexts [46]. In Cambodia, people with a disability have reported incidents where they have been made to pay extra fees for mobility devices, or have been denied access to public transport because of the extra space required for the devices [47,48]. Kleinitz et al. [47] noted in 2012 that people in wheelchairs often need to be lifted into motorcycles, rickshaws and buses, which may discourage individuals from using such methods of transport to access health services or medical facilities. While a public bus system has been introduced in Phnom Penh since Kleinitz et al.’s report, and people with a disability can travel for free, the same issues were observed during the pilots and reported by participants as a problem for them more generally.

As an alternative to use of standard public transport services, buses and taxis may be hired privately, but they do not have the space required to accommodate wheelchairs and the cost may be prohibitive [49]. Overall, the quality of roads in Cambodia is not high, and in Phnom Penh the condition of the footpaths and the nature of the traffic are not conducive to travel by people with a disability.

In the first draft of the JAT, Cambodian examples were used in questions based around the United Nations Enable’s *Accessibility for the disabled: a design manual for a barrier free environment*. However, it was identified in discussion with HI that the design manual was too complex to use, as it required measurements to be made and utilised jargon for features of the built environment. Consequently, following team consultation, the JAT was simplified and divided into distinct sections:

- Getting to the transport stop;
- Intersections and crossings;
- Accessing the transport stop;
- Access to the vehicle and boarding;
- Access to formal stops;
- Public transport staff; and
- After boarding to the destination.

Feedback was again provided by the team, and the questions were further simplified and condensed. The JAT was translated into Khmer and feedback was sought from representatives of people with a disability in Cambodia. This resulted in further redrafting in preparation for the pilot phases. Figure 3 presents sample pages (in English) from the resulting draft JAT.

### Pilot testing

Three pilot studies were conducted on the streets and buses of Phnom Penh to test the procedural and conceptual aspects of the JAT. The participants were people with a disability recruited by HI. No minors participated in the on-road aspects of the pilots, and the participants were accompanied by assistants who were HI staff or volunteers. An enabling approach allowed the people with a disability, assistants, HI and QUT team members who participated to freely critique aspects of the initial JAT and offer suggestions on how the tool could be improved. While the main aim of the research was to test how the tool worked in practice, valuable insights were also gained about the types of barriers to access experienced.

The first pilot was conducted over two days (12 and 13 February 2015). Two QUT team members visited to assist with finalisation of preparations and to participate in the pilot. The first day was devoted to training and induction for participants, student assistants, and personnel from HI and QUT. On the second day, five groups were formed, varying in size but containing at least one assistant and one HI staff member. The participants included one person using a wheelchair, one person with visual impairment and others with physical impairment, each with an assistant. An HI staff member with technical expertise accompanied the groups to evaluate the design and effectiveness of the JAT.

The groups travelled by *tuk-tuk* from the HI office in Phnom Penh to the designated start point. The route included a bus ride, a walking or wheelchair to a supermarket, and return to the HI office for group discussion and a feedback session. The feedback focus group session was conducted in Khmer and feedback was documented and translated into English.

The second pilot was undertaken on 28 and 29 May 2015. Ten groups (eight during the day and two at night) were formed from the nine participants (i.e. one participated in both day and night groups). Again, the first day involved training of assistants and an induction process for all participants. The JAT trial followed two different routes, using two bus lines, and included a night journey. The nine participants included one person with a disability in a wheelchair, two people with visual impairment, and others with physical impairment. In response to findings from the first pilot (see next section), the second trial ensured there were shorter verbal discussion sessions between the participant and their assistant on the route. Instead, the assistant recorded the views of the participant rather than verbal comments being transcribed with pen and paper as had occurred during the first trial. Additionally, participants were provided with cameras to personally capture what they perceived to be barriers to access. The longer summing up process was left to the end of the day at the debriefing session (this was before the night journey, which was only partially completed due to non-arrival of the bus). Another recommendation from the first pilot, to ensure participant responses were not ‘overwritten’ by the comments of assistants, was actioned by transcribing feedback verbatim from participants and having a debriefing session separate from the assistants.

There were seven participants in the third pilot, conducted on 30 September and 1 October 2015. They included people with physical disability, sensory impairment, low vision, and speech impairment. Six daytime groups and two night-time groups were formed. One HI staff member and one assistant accompanied each group. Two bus lines were used for both the day trips (three groups each) and night trips (one group each).

The focus groups at the end of each pilot solicited feedback from participants on the journey, process issues in use of the JAT, and recommendations for improvements to the tool and the process. Observations made by HI and QUT team members were collated and discussed in person, via Skype and email. Iterative changes were made to the tool and the process over the course of the pilots.

## Results

The primary focus of the research was on participants’ views on the tool; however, they gravitated towards commenting on the barriers to access they encountered, and needed to be refocused regularly. Some of their comments on the barriers appear later in this section.

### First pilot

Some participants commented that assistants attempted to reinterpret their comments about their experiences as they were using the JAT. Although they acknowledged that this was done with good intentions, it was suggested that this tendency had an adverse effect on the feedback process. This issue was further discussed by HI and QUT team members who had participated in the pilot. The following recommendations were made by participants about the JAT itself:

- There is a need to add and clarify terms, especially technical terms for road environment features;
- Photos should be added to provide examples of some of the roadway features referred to;
- More questions are needed to cover issues on the bus;
- It is advisable to change negative questions to positive, since the yes/no response pattern in Khmer is opposite to that in English for negative questions, which means the translated answers are ambiguous;
- It might be worth ‘grading’ the amount of accessibility on a path, since most paths have accessibility problems, but some are much worse than others.

In addition, there were recommendations about the JAT process:

- There was a lack of clarity about the relative role of participants and assistants in identifying barriers to access; in practice, this was due to the difference between the perceptions of participants from their own perspective as travellers with a disability, and the observations made by assistants on behalf of people with a disability in general regarding barriers and access;
- Related to the above, the briefing and training of assistants needed to be enhanced;
- The photocopied version of the JAT was large and unwieldy (A4 sheets of paper in a folder), so the format needed to be changed to make it easier to use when travelling;
- There was a need to conduct pilots at night, as there are some significant additional challenges not apparent during daylight hours;
- Different impairments have different needs, so having participants with different impairments provides multiple perspectives on barriers to access, with the complication that there are different solutions for different impairments;
- The possibility of two tools was floated – one for use by participants, the other by support persons or professional staff, similar to the way road safety audits are conducted.

These recommendations were taken into account by the QUT team in the revision of the JAT and the JAT process in preparation for the second pilot, although not all recommendations were implemented for reasons of policy, process or practicality. For example, the development of two tools was acknowledged as having potential value in the future, but the focus on an inclusive approach meant that the pilots should be restricted to a JAT that can be used by people with a disability. The recommendations about clarification of technical terms and provision of photos were implemented, the JAT was shortened and simplified (although extra bus items were added) and changes were made to clarify the role of the assistants in relationship to facilitating participants to record their views and experiences rather than capturing the impressions of the assistants. In order to bring the perceptions of the participants into the foreground, the JAT instructions asked the assistants to include verbatim comments from participants about barriers.

### Second pilot

One outcome of the change in approach in the second pilot was a more personal experiential account of the journey from the perspective of the participant, rather than simply identifying barriers to safe journeys. It was in line with this philosophy that cameras were provided to participants. However, some participants commented that their assistants were still offering too much help in recording information and intervened too much in addressing the physical barriers encountered in the journey. Some of the assistants requested more support on how they could facilitate the pilot without providing unneeded interpretation or assistance.

As in the first pilot, participant feedback was integrated into discussions between HI and the QUT team on their observations. The following feedback was provided about the tool:

- There was too much repetition of questions on each part of the route, e.g. there were several road sections with footpaths on the way to the bus stop, and the same questions were asked for each section about barriers – need to avoid this if possible;
- There were problems with translating some words, where different English words were translated into the same single word in Khmer – need to find a way to clarify differences in meaning;
- The JAT refers to ‘formal’ and ‘informal’ bus stops, but the participants and assistants did not know what was meant by this distinction;
- Some items need to take account of the impairment, e.g. visual impairment means that barriers are experienced rather than seen.

With respect to the JAT process, the following recommendations were made (noting there was some overlap with the points above):

- Need to distinguish between perception of participants and opinion of assistant re barriers;
- The time taken to conduct the JAT needs to be shortened.

On the basis of the recommendations, further changes were made to the JAT. The most significant was division of each journey into four discrete parts: trip from origin to transport stop; the transport stop itself; boarding and travelling on the vehicle; and from dismounting point to destination. Participants were encouraged to make a more global assessment at the end of each of these parts, rather than providing a detailed breakdown. In addition to shortening the task, it was reasoned that this process would provide sufficient information for a road environment expert to review the treatment needs of the trip components later. This version of the JAT is provided as supplemental material.

### Third pilot

At the beginning of the focus group session the participants discussed feedback on general organisation and the updated questionnaire. Most participants agreed the questionnaire was not too repetitive, although some terminology required clarification and some questions were difficult to understand. A participant with an auditory impairment suggested that more time was needed to communicate the questions and instructions in sign language. Another participant commented that assistants may have provided too much physical support at times, leading to less accurate identification of journey barriers. Some participants commented on the length of the trial and discomfort experienced. For example, the walking or wheelchair section to the beginning of the pilot trip was too long, answering questions was difficult due to standing in the sun for long periods, and traffic noise made it hard to hear the assistant. Two of the groups suggested that general comments could be gathered during the journey but formal questions could be asked later to avoid standing in hot, noisy and dangerous locations. The night journey group felt it was useful to understand how conditions changed in low light and how access was made more difficult due to vehicles parked on the sidewalk and an increase in street vendors after dark. The comments in Pilot 3 regarding excess assistance by the HI staff indicate that more training is required but also that it is difficult for staff to override an empathic response to difficulties and barriers experienced by people with a disability.

These items and the overall feedback were discussed by HI and the QUT team members, leading to the following comments/recommendations about the tool:

- The JAT is noticeably improved, in particular it is now less repetitive;
- Some terminology needs clarification, in this case the difference between ‘formal’ and ‘informal’ road crossings.

With respect to the JAT process, the following comments were made:

- Assistants can provide too much help, so the experience is different to what would be experienced by a person with a disability travelling alone;
- There are time and exposure issues (sun, heat, noise, pollution) when there is a stop to record information;
- The night trip is worthwhile as it changes the conditions – there are more vehicles parking on the sidewalk, as well as vendors, and many barriers visible in the daytime are hard to see at night, e.g. potholes.

Notably, the issue of the role of assistants was raised again. Concern about exposure to the elements had not previously been mentioned.

### Identification of barriers

Although the formative evaluation was about the tool and the audit process, participants generated rich information on the barriers to access they observed on their journeys. Following the third pilot, some informal consultation was undertaken with the authorities responsible for the road environments and transport services in Phnom Penh. They commented that the insights from the JAT drew attention to issues they had not been fully aware of, and could provide a basis for prioritising improvements to the road environment and transport services.

## Conclusions

During the drafting and piloting of the JAT, its content and implementation process were refined, with people with a disability playing a key role in modifications. Issues of translation and terminology were addressed relatively early, suggesting that adaptation to a new context can be achieved through strategic use of iterative pilots. Although the structure of the journeys took longer to resolve in order to reduce the amount of time spent recording experiences of the journey, the iterative changes to the process means that journeys can be structured more effectively from the start when new projects are undertaken.

People with a disability are often viewed through stereotypes of inability and dependency. Hence, the interpersonal dynamics of the JAT journeys proved more difficult to address, specifically the predisposition of the assistants to speak on behalf of the person with a disability or interpret their views. They tended to provide too much help in the form of comments about barriers and generalisations beyond the experience of the people with a disability who participated. Previous research in Cambodia provides a possible cultural explanation for this, finding that it is the norm to express pity or compassion for people with a disability, using terminology associated with the Buddhist concept of earning merit [50]. As a consequence, the concepts of empowerment and inclusivity in relation to disability that are promoted through the UN Convention on the Rights of Persons with a disability may not have been understood by the assistants, which may have contributed to their tendency to be overly helpful. The training process for assistants is an important part of the JAT and this content was refined over the three pilots. Regardless, it is likely that the need to ensure that JAT privileges the voice and experiences of the person with a disability rather than interpretations through the lenses of the assistants will require proactive management by the organisers of future JAT projects. A template for future use of the JAT has therefore been developed that includes this important dimension.

Potential limitations of the approach were the recruitment of participants and assistants through HI networks, and the imposition of a particular journey and structure on participants. While these constraints were necessary given the formative nature of the evaluation, at a later stage it is envisaged the participants would define the journey for which the JAT is used, including the way it is segmented, e.g. by mode. Participants’ familiarity with the journey might avoid the need for assistants, and segmentation by mode might reduce repetition.

Although it was beyond the scope of this research, there are two further steps that are important if use of the JAT is to be truly effective in achieving real change to facilitate safe access for people with a disability to services including health care, employment or education. The first is use of the JAT results to advocate for road environment and transport improvements that are disability inclusive. Presumably, this would involve visual presentation of the main messages about barriers to access identified, and promotion of the desired actions to the agencies concerned. The second step extends this approach, and concerns using the JAT as a potential tool for meaningful communication and engagement with the relevant agencies that enables people with a disability to enjoy full community participation and citizenship. This positive approach is very different from adversarial approaches. The JAT has potential as a powerful evidence-based advocacy tool used by individuals, families, service providers or disabled persons’ organisations. The version of the JAT used in the third pilot is provided as supplemental material and can be used under a Creative Commons licence, with appropriate attribution, for non-commercial purposes.

## Paper context

People with a disability in low- and middle-income countries often need to travel to access health services employment, education, shops and social contact. However, the physical environment and traffic present significant barriers and risks that have been little studied. The combination of access audits and road safety audits into an acceptable and adoptable tool offers potential for people with a disability to identify access issues on journeys they take, and enable them to advocate for change.
